# Grounding clinical and cognitive scientists in an interdisciplinary discussion

**DOI:** 10.3389/fpsyg.2013.00630

**Published:** 2013-09-19

**Authors:** Giovanni Ottoboni

**Affiliations:** ^1^Department of Psychology, University of Bologna, BolognaItaly; ^2^Centro di Psicologia e Psicoterapia Funzionale Integrata, TriesteItaly

**Keywords:** embodiment, grounded cognition, embodied clinical perspectives

## Abstract

In most clinical approaches the body receives little attention. In cognitive science, in contrast, the embodied and grounded perspective, which emphasizes the importance of the body, has been intensively explored over the last decade. The present article aims to engage theorists of embodied cognition and clinical experts in a discussion encouraging them to consider the insights that may arise from each other’s approaches. In a review of the cognitive and clinical literature substantial overlap is revealed between cognitive and clinical domains.

The interpretations academics and professionals offer for psychological disorders are not unanimous. Traditional therapeutic approaches explain that psychological disorders arise from irrational beliefs and illogical thought patterns or from unresolved emotional conflicts (e.g., Zeig, 1997; Sutker and Adams, 2001). Within these perspectives, an even wider range of treatments is offered. Some treatment options are targeted at changing learned behaviors, others are aimed at reshaping old attachment styles, others work on memories arising from relations occurring within the original family system, and others involve medical psychosomatic concepts. Few approaches to clinical treatment, however, are able to provide a comprehensive and integrated theoretical account of all human expressions (Palmer and Woolfe, 2000).

Alongside such views, the connection between the body (and bodily states), cognition and emotion is theoretically accepted (Van Oudenhove and Cuypers, 2010). According to some therapeutic approaches (Totton, 2003), and even in some early works in psychodynamic theory – later studied in depth by other psychoanalysts such as Perrin (2010) – the body plays an important causal role in the development of mental disorders. From a more body-oriented perspective it is argued that the body is included in all processes involved in self-awareness (Segal et al., 2002). Röhricht and colleagues (Röhricht and Priebe, 2006; Röhricht et al., 2013) provide congruent evidence. For example, the authors specifically report on the positive effects of the bodily therapy in two separate groups of schizophrenic and depressed patients. They showed that the negative, depressive symptoms decreased more than in controls. The authors also report that bodily techniques are effective for treatment of mental disorders among patients who do not respond to traditional talking therapies, e.g., somatoform disorders/medically unexplained syndromes, post-traumatic stress disorder (PTSD), anorexia nervosa, and chronic schizophrenia (Röhricht and Priebe, 2006; Röhricht, 2009). Also for medical practice, a number of clinical studies support that bodily therapies have positive impacts in pathological conditions (Moyer et al., 2004; Tsao, 2007).

One of the approaches that is presently attracting the attention of researchers and professionals by claiming a full integration between the bodily and the psychological aspects is the Mindfulness approach. Mindfulness is described as “a process of regulating [clients’] attention in order to bring a quality of non-elaborative awareness to current experience and a quality of relating to one’s experience within an orientation of curiosity, experiential openness, and acceptance” (Bishop et al., 2004, p. 234). In recent years, a number of studies have investigated the both the psychological and the physical modulations that can be achieved when people reach certain states of mindful awareness (Grossman et al., 2004; Michalak et al., 2010). Mindfulness techniques have been used to enhance self-observation from inner and outer perspectives. The mindfulness approach represents a *third-wave* for many clinical and non-clinical treatments because it prepares people to respond functionally and consciously to their environment (e.g., Boyle, 2011).

In recent years, the connection between what is expressed and conveyed by the body and cognitive, functional and emotional expressions has received renewed interest from a wide range of neuroscientists. Damasio (2005) approached the mind-body link directly by proposing that somatic markers are intimately related to thinking and decision-making. In a similar manner, other scholars have suggested the existence of a *gut–brain/brain–gut axes* (Mayer, 2011). Gut microbes appear able to transmit information directly to the central nervous system (CNS), communicating many of the changes that occur in the gut. Through this communication pathway, the CNS can identify the presence of pathogens in the gut lumen and activate appropriate response mechanisms. It seems that the level of intestinal microbiota and inflammation markers have a role, as for example, in the depression states (Bested et al., 2013; Rawdin et al., 2013).

From a cognitive perspective, the body–mind coupling has been at the center of scientific discussion in neuroscience for many years. In the middle of the last century, Yarbus (1967) described the importance of the muscular eye movement for vision and visual attention. Similarly, Liberman et al. (1967) proposed that language comprehension is inseparable from language production. More recently, a group of neurons was discovered in the monkey premotor cortex (di Pellegrino et al., 1992). The neurons able to produce electrophysiological spikes that have a similar pattern regardless of whether the monkey is executing an action or observing the same action but performed by the experimenter (i.e., grasping some monkey food). The *double-firing* property has led to this group of neurons to be termed *Mirror Neurons* (Gallese et al., 1996). When evidence of a similar system was reported in humans (Mukamel et al., 2010), the connection between body and mind entered centrally in the domain of psychology. The mirror neuron network serves as an automatic and involuntary system that is strengthened by links, such as motor expertise, between the action observer and the action performer (Castiello et al., 2009). Dysfunctions within this reverberating network are also considered to be a basis of empathic deficits associated with autistic spectrum disorder (Williams, 2008). In last years, the human mirror neuron system network has been found to respond to various stimuli, including action words (D’Ausilio et al., 2009) and pain-related stimuli. Avenanti et al. (2005) reported higher levels of activation in the brain area that principally controls hand movements when participants watched a video clip of a needle piercing a person’s hand than when participants watched a neutral video in a control condition. However, in some cases, the mirror neuron network has been used to explain even the mechanisms of empathic and emotional resonance that come into play in therapeutic settings (Berrol, 2006; Gallese et al., 2007; Schermer, 2010), as well as cultural, social, and psychodynamic interactions (Vanderwert et al., 2012).

With the growing evidence for the influence of the body in the control of cognitive processes, a new perspective (e.g., Varela et al., 1991; Borghi and Pecher, 2012), known as Embodied Cognition (EC) has developed. Recently, theorists have argued that mind-body influences are related to bodily states as well as to the physical and bodily experiences people have (Fischer, 2012). Along this vein, Barsalou (2008) suggested that the use of the concept of *grounding* is preferable to embodiment because the former includes concepts relating to simulation that are able to occur even when the action actor and the action observer do not share the same action motor control. This would be the case if a patient suffering apraxic were to engage in conversation about a pen. The patient would be able to name and describe the pen and provide relevant information about it, such as where it can usually be found, but would not be able to perform actions with the pen, such as write with the pen. In this way, aside from the boundaries of the body, the concept of *grounding* relies more on the effectiveness with which physical experiences interact with cognitive processes (e.g., Symes et al., 2008; Eder and Hommel, 2013).

Aside from the growing scientific and clinical evidence supporting the *grounded* body–mind interconnection, what appears missing from both fields is a proper translational process that integrates the scientific and clinical domains. Regarding clinical psychology, the opinions of academics and professionals differ greatly: there are cases in which *grounding* the therapeutic process in bodily terms using movements, posture, and physiological indexes is neglected or it is used only metaphorically (Sensky et al., 2007). There are examples of professionals who commit to theater, yoga, and dance the bodily healing aspect. Such a process of devolution is necessary when it is not possible to theoretically integrate such aspects into the existing theory (Palmer and Woolfe, 2000). On the other hand, there are health care professionals who are accustomed to working only with the physicality of the body and who find themselves unprepared for dealing with emotional and psychological aspects that arise during treatment.

One relatively new clinical approach that integrates bodily and psychological aspects is the Neo-Functionalism (NF) approach (Rispoli, 2008; Ottoboni and Iacono, 2013). The NF approach was developed from the body-centered perspectives. The involvement of the body within the therapeutic setting has generated two kinds of advantages: it provides the clients with the opportunity to communicate their psychological states directly without limits and it provides the therapist with the opportunity to get deeply in touch with the clients’ emotions and expressions. According to this view, indeed, the body conveys and communicates the individual’s psychological****states as it receives feedback from outcomes of physical actions. Body movements and facial expressions, as well as the contextual information have been indicated as influencing psychological states and activities, such as memory, predisposition, and decision-making (Strack et al., 1988; Hatfield et al., 1992; Craig, 2002; Dijkstra et al., 2007) and pain perception (Avenanti et al., 2005).

In the process of clinical evaluation and treatment, NF considers all life experiences people have had according to the experiences’ *grounded* and embodied aspects. The core concept of the NF approach relies on a discrete group of life instances whose experiences affect cognitive functioning and expressions (Rispoli, 2008; Ottoboni and Iacono, 2013). They are claimed to be universal and are called Basic Experiences of the Self (BEsS) to denote the strict connection between two concepts, the Self and *grounded* experiences. Indeed, the connection between behaviors (independently of their sane or deviant forms), the neurological background (intact or damaged), the social context (read social experiences) and the development of the Self has recently arisen a number of interesting debates within the scientific community (see, for example, Blanke and Metzinger, 2009; Brugger et al., 2013; Reed and McIntosh, 2013).

The way individuals experience each Basic Experience of the Self (BES) produces cognitive, emotional, physiological, and postural-related outcomes. Each time the same BES is experienced the individual stores a memory of the outcomes of the experience, matches them with past experiences and uses them to create expectations for the future (see Logan, 2002; see also Mancia, 2006 for a psychodynamic perspective). If the BES is experienced positively, memories are formed and are made available later for dealing with novel situations. In contrast, if the response to the BES is maladaptive, it may generate a sense of inability to deal with novel situations (Rispoli, 2008; Ottoboni and Iacono, 2013). Each BES may be experienced several times during the lifespan. The averaged mode in which each BES is experienced determines the manner in which memories of the BES are stored. The account that only the repeated outcomes of the same BES can modulate the behavior highlights that such a *grounding* process of memory development is not a point-to-point process; it required time either to form the maladaptive responses or to develop positive responses. Hence, if a large number of BEsS are experienced negatively, the individual’s responses to environmental demands will be poor because of a low level of resilience (Rothschild, 2000); indeed, the higher the functioning, the more adaptive the responses.

The NF interpretation of the word *functioning* is the same that *grounding* theory provides: each bodily expression (i.e., function) comprehends cognitive, emotional, postural, and physiological features (see also Hatfield et al., 1992). In line with this view, a depressed demeanor expresses a number of cognitive, physiological, and emotional features as well as bodily postures that must all be taken into account during therapeutic treatment (e.g., Michalak et al., 2009). By considering human expression as complex in nature, the NF therapist is able to find the most appropriate manner for interacting with the client. If one manner of expression is inappropriate, another manner may be pursued, and, as suggested by Röhricht (2009) in referring to body-centered techniques, unexpected results can be achieved. Let us consider, for example, a person suffering ruminations. The patient may be very careful and skilled at identifying appropriate verbal responses to the therapist’s requests. In such cases the therapeutic process could be made difficult. A way to achieve treatment results in such cases would be for the therapist to use the physical channel. The therapist must thus work on the client’s body (Ottoboni and Iacono, 2013) with calm and wide hand-on massages. Indeed, the focus of this therapeutic technique would be to calm the patient’s thoughts and let the therapeutic process to begin.

The healing outcomes are achieved by helping the client to re-experience the BEsS that were not positively experienced in the past. The process is intensive because, by using a *grounding* approach, the therapists tend to re-create the same physical and emotional conditions the patient experienced when the trauma begun. Mental thoughts, verbal expressions and body-related experiences are used in a combined fashion. The clients could, for example, be asked to lie down on the ground as they did in childhood, to wander in the room and express their feeling during the walk, or to vocalize with pre-verbal utterances the psychophysical sensations that therapeutic hand-on messages have made emerge. To repeat, these techniques are mainly used to make the patients emotionally regress to the specific moment of their past, because, in this way, the client may discharge the old memories and form new ones from the experience just-lived (Rispoli, 2008; Ottoboni and Iacono, 2013).

The change in the patient that the NF therapist aims to achieve concerns the attempt to reconstruct the harmonious organization of the Self as it was in the womb. Even if such a concept could be criticized for not accounting for the gestational period, the fetus could potentially experience problems and disease, premonitory of future functioning. I personally consider that the harmonious state indicates a general and natural state toward which everybody is inclined to experience. Using a Mindfulness concept, it could be claimed that such a harmonious state is the state of acceptance of internal and external changes (Grossman et al., 2004). This state involves a calm and relaxed state of mind.

## CONCLUSION

A growing number of studies have shown that human cognition, emotions, and behaviors have embodied features, or as Barsalou (2008) has preferred to describe, *grounded* features.

However, this knowledge still remains in the research domain and is rarely applied in clinical practice. An attempt to translate *grounded* evidence in clinical practice has been introduced****in a recent paper. Bedford (2012) theorizes that the visual component of perception can cure a number of medical symptoms by affecting the immune system. Vision, however, is controlled and modulated (Rizzolatti et al., 1994) by the motor system, as in the case of visual awareness studies demonstrating that the motor plan moderates perceptions of plan-congruent objects (Symes et al., 2008), or the reaching of a target in absence of visual awareness (Binsted et al., 2007). Interestingly, the visual information concerning the body is integrated and combined in several areas in the brain with information coming from the other senses too (see Blanke, 2012 for a review). As soon as the visually based information, mainly defining a map-like representation of body (e.g., Tessari et al., 2010), are integrated with kinesthetic and vestibular information, a body-based sense of self takes place (Blanke and Metzinger, 2009).

In sum, it appears that the approaches that effectively integrate cognitive and motor aspects of human behavior are few in number. One such approach is the NF approach (Rispoli, 2008; Ottoboni and Iacono, 2013). By working simultaneously on imagery-related techniques, verbal and bodily techniques (such as hand-on massages or body movements), the NF approach accounts for all aspects of human expression from a *grounded* perspective. During treatment, past experiences, which generate actual psychological states, are recalled and experienced again physically within a clinical setting.

However, the *grounded* assumptions, as well as the *grounded* techniques, must be tested thoroughly. As this review demonstrates, embodied cognition and *grounded* clinical approaches are still far from integrated with each other. Research exploring the effects of *grounded* therapeutic approaches should be a priority for scientists interested in understanding human behavior and its therapeutic treatments.

Scientific evidence suggests that embodied cognition could be very useful for achieving such an aim. Equally, studies dealing with embodied cognition could take advantage of what is known in clinical settings.

## Conflict of Interest Statement

The author declares that the research was conducted in the absence of any commercial or financial relationships that could be construed as a potential conflict of interest.
